# Functional Analysis of p21^Cip1/*CDKN1A*^ and Its Family Members in Trophoblastic Cells of the Placenta and Its Roles in Preeclampsia

**DOI:** 10.3390/cells10092214

**Published:** 2021-08-27

**Authors:** Nina-Naomi Kreis, Alexandra Friemel, Lukas Jennewein, Samira Catharina Hoock, Anna Elisabeth Hentrich, Thorsten Nowak, Frank Louwen, Juping Yuan

**Affiliations:** 1 Obstetrics and Prenatal Medicine, Department of Gynecology and Obstetrics, University Hospital Frankfurt, J. W. Goethe-University Frankfurt, Theodor-Stern-Kai 7, 60590 Frankfurt, Germany; Alexandra.Friemel@kgu.de (A.F.); Lukas.Jennewein@kgu.de (L.J.); SamiraCatharina.Hoock@kgu.de (S.C.H.); AnnaElisabeth.Hentrich@kgu.de (A.E.H.); Louwen@em.uni-frankfurt.de (F.L.); yuan@em.uni-frankfurt.de (J.Y.); 2 Medical Practice for Gynecology and Obstetrics, Mainzer Landstr. 265, 60326 Frankfurt, Germany; info@dr-nowak.net

**Keywords:** p21^Cip1/*CDKN1A*^, trophoblasts, preeclampsia, hypoxia, trophoblast organoids, fusion

## Abstract

Preeclampsia (PE), a gestational hypertensive disease originating from the placenta, is characterized by an imbalance of various cellular processes. The cell cycle regulator p21^Cip1/*CDKN1A*^ (p21) and its family members p27 and p57 regulate signaling pathways fundamental to placental development. The aim of the present study was to enlighten the individual roles of these cell cycle regulators in placental development and their molecular involvement in the pathogenesis of PE. The expression and localization of p21, phospho-p21 (Thr-145), p27, and p57 was immunohistochemically analyzed in placental tissues from patients with early-onset PE, early-onset PE complicated by the HELLP (hemolysis, elevated liver enzymes and low platelet count) syndrome as well as late-onset PE compared to their corresponding control tissues from well-matched women undergoing caesarean sections. The gene level was evaluated using real-time quantitative PCR. We demonstrate that the delivery mode strongly influenced placental gene expression, especially for *CDKN1A* (p21) and *CDKN1B* (p27), which were significantly upregulated in response to labor. Cell cycle regulators were highly expressed in first trimester placentas and impacted by hypoxic conditions. In support of these observations, p21 protein was abundant in trophoblast organoids and hypoxia reduced its gene expression. Microarray analysis of the trophoblastic BeWo cell line depleted of p21 revealed various interesting candidate genes and signaling pathways for the fusion process. The level of p21 was reduced in fusing cytotrophoblasts in early-onset PE placentas and depletion of p21 led to reduced expression of fusion-related genes such as syncytin-2 and human chorionic gonadotropin (β-hCG), which adversely affected the fusion capability of trophoblastic cells. These data highlight that cell cycle regulators are important for the development of the placenta. Interfering with p21 influences multiple pathways related to the pathogenesis of PE.

## 1. Introduction

Pregnancy can be affected by various health problems, of which preeclampsia (PE) is the most common. PE is a multisystemic gestational disease with a global prevalence of up to 8% [1]. It is characterized by concurrent hypertension and proteinuria or any other sign of end organ damage including liver or brain, occurring after 20 weeks of gestation [2]. PE is a consequence of diverse pathophysiological processes linked to maternal endothelial dysfunction and systemic inflammation, which can result in multiorgan failure, if the fetus and placenta are not delivered [1,2,3]. It can be subdivided into an early-onset (<34th week of gestation) and a late-onset (≥34th week of gestation) form [4]. Since early-onset PE mainly originates from the placenta, it is also referred to as placental PE, where it is tightly associated with defective trophoblast invasion and inadequate spiral artery remodeling, while late-onset PE is rather linked to preexisting maternal risk factors like obesity [5]. This classification has prognostic importance, since early-onset PE entails a greater risk of maternal and fetal complications [6]. PE is often complicated by the HELLP syndrome, which is an acronym for hemolysis, elevated liver enzymes, and low platelet count [7]. Despite intensive research in the last decades, prevention and treatment options are very limited. Delivery is currently the only definitive treatment for PE patients. Consequently, it remains one of the leading causes of maternal and perinatal mortality and morbidity, and is associated with increased rates of cardiovascular and metabolic diseases in later life [1,8].

Defective placentation is considered to be causative for the development of pregnancy-related disorders like PE, fetal growth restriction [9], and the HELLP syndrome [10], suggesting that trophoblasts, which constitute the major placental cell type, are deficient and the origin of gestational diseases. The human placenta is a rather complex organ structured as villous trees consisting of an inner layer of proliferative progenitor cells, called villous cytotrophoblasts (CTBs). On one hand, these CTBs further differentiate and then fuse into the non-proliferative and multinucleated syncytiotrophoblast (STB), responsible for nutrient exchange and hormone production [11]. Of importance, high attention is paid to a small portion of villous CTBs, so called “fusing cytotrophoblasts” (fCTBs), which are characterized by a marked reduction in E-cadherin expression during the differentiation process to the STB [12,13] and ready to fuse to the STB, thus, also referred to as intermediate CTBs [14]. On the other hand, CTBs grow into the proliferative cell columns and differentiate to an invasive and growth-arrested phenotype, termed extravillous trophoblasts (EVTs) [11]. CTBs are therefore considered as progenitors for the STB as well as EVTs [15]. EVTs invade into the maternal decidua and colonize the lumen of spiral arteries, which are remodeled for sufficient blood supply to the embryo [16]. PE is associated with profound cellular dysfunctions including impaired placentation, deregulated proliferation and differentiation manifested by shallow EVT invasion with incomplete spiral artery remodeling, defective fusion of CTBs, increased STB microparticles due to deregulated apoptosis, and a hypoxic environment resulting in elevated oxidative stress, angiogenic imbalance, and inflammation at the maternal–fetal interface [1,17,18,19,20].

The rapid development of the human placenta requires strictly regulated and coordinated cell proliferation and differentiation processes, crucial for tissue homeostasis [21]. While a successful placentation depends on precise trophoblast regulation, the roles of cell cycle regulators are understudied in the human placenta. p21^Cip1^, encoded by the gene *CDKN1A* (cyclin-dependent kinase inhibitor 1A), is a pivotal broad cell cycle regulator with heterogeneous roles and the founding member of the Cip/Kip (cyclin-dependent kinase interacting protein/kinase inhibitory protein) family including p27^Kip1^ (*CDKN1B*) and p57^Kip2^ (*CDKN1C*) [22]. Interestingly, these regulators are evolutionally highly conserved and able to substitute each other [23]. Apart from its role in cell cycle regulation including mitosis, p21 is involved in cell differentiation, reprogramming of induced pluripotent stem cells, transcription, DNA repair, migration, apoptosis, autophagy, and the onset of senescence [24]. p21 acts either as a positive or negative regulator of the same pathway, often related to its cytoplasmic localization, expression level, and posttranslational modifications [23,24]. Since p21 participates in a diversity of cellular activities in cancer cells and stem cells, it is reasonable to assume that p21 is also multifunctional in placental trophoblasts coordinating various cellular processes. In fact, we have recently shown that the deficiency of p21 lowers the migration and invasion capability of cancer and trophoblastic cells [25]. In the present work, we investigated the expression and localization of p21 and its family members in the placenta, and their potential involvement in placental development and the pathogenesis of PE.

## 2. Materials and Methods

### 2.1. Placental Tissue Collection

This study was approved by the Ethics Committee at the University Hospital (reference number: 375/11), Goethe-University Frankfurt. Written informed approval was obtained from all donors. PE was diagnosed as an occurrence of hypertension after 20 weeks of gestation with a blood pressure ≥140/90 mmHg and proteinuria with ≥300 mg in 24 h. The HELLP syndrome was defined as the presence of hemolysis, elevated liver enzymes, and thrombocytopenia (low platelet count <100,000/µL). The reasons for delivery of early-onset control groups were PE-irrelevant like breech presentation, premature rupture of membranes, premature placental abruption, non-reassuring fetal heart rate, or umbilical cord prolapse. Tissue samples were taken from placentas within 30 min post-delivery, formalin-fixed and paraffin-embedded (FFPE) for immunohistochemistry staining (IHC), or frozen immediately in liquid nitrogen for mRNA and protein extraction, which were stored at −80 °C until usage. Clinical information of all participants is shown in (Table 1, Table 2, Table 3 and Table 4). For IHC staining, Prof. Dr. Qi Chen, Department of Obstetrics and Gynecology, University of Auckland, and Fudan University, Shanghai, China, kindly provided us with six first trimester placental FFPE samples (six to nine weeks of gestation, age 20 to 33 years). The sample collection was approved by the Ethics Committee of the Hospital of Obstetrics and Gynecology of Fudan University (reference number 2018-62), China. Written consent was obtained from healthy donors undergoing elective surgical terminations of pregnancy. For trophoblast organoid formation, primary villous cytotrophoblasts or placental mesenchymal stem/stromal cells were isolated from first trimester placentas. Written informed consent was obtained from patients undergoing elective terminations of normal pregnancies (seven to 12 weeks of gestation, age 25 to 41 years) at the Medical Practice for gynecology and obstetrics led by Dr. Thorsten Nowak with ethical approval from the Ethics Committee at the University Hospital, Goethe-University Frankfurt (reference number 19-455).

### 2.2. Formation of Trophoblast Organoids from Human First Trimester Placental Tissue

The protocol was adapted from Sheridan et al. [26] with some modifications. In brief, to obtain trophoblast-enriched cell suspensions, villi from first trimester placental tissue were washed with PBS (Thermo Fisher, Waltham, MA, USA) containing 1% penicillin/streptomycin (Sigma-Aldrich, Taufkirchen, Germany), further minced into small pieces and sequentially digested with 0.2% trypsin/EDTA in PBS, then with 1.0 mg/mL collagenase V (Sigma-Aldrich, Taufkirchen, Germany) in PBS containing 0.1% BSA (Carl Roth, Karlsruhe, Germany). The digestion steps were performed in a shaker with 120 rpm for 5 min at 37 °C. Both digestion steps were stopped with Ham’s F12 medium (Life Technologies, Carlsbad, CA, USA) containing 20% fetal bovine serum (FBS) (Biowest, Riverside, CA, USA), filtered with a 100 μm cell strainer (Corning, New York, NY, USA), pooled and washed with PBS. Erythrocytes were lysed by the addition of lysis buffer (155 mM NH_4_Cl, 10 mM KHCO_3_, 0.1 mM EDTA) [27], incubated for 10 min at 37 °C, centrifuged (1000× *g*, 5 min), and washed twice with PBS. Cells were plated onto 24 well Corning Costar ultra-low attachment plates (Corning; Berlin, Germany) or embedded in Matrigel (Trevigen^®^, Gaithersburg, MD, USA). The trophoblast organoid medium (TOM) contained Advanced DMEM/F12 supplemented with 1× N2 (Life Technologies), 1× B27 (Life Technologies), 100 µg/mL primocin (Invivogen, San Diego, CA, USA), 1.25 nM *N*-Acetyl-l-cysteine (Sigma-Aldrich, Taufkirchen, Germany), 2 mM L-glutamine (Life Technologies, Carlsbad, CA, USA), 1 mM A83-01 (Tocris, Bristol, UK), 1.5 µM CHIR99021 (Selleck Chemicals Llc., Houston, TX, USA), 50 ng/mL EGF (epidermal growth factor) (PeproTech, Rocky Hill, NJ, USA), 100 ng/mL R-spondin 1 (PeproTech, Rocky Hill, NJ, USA), 100 ng/mL FGF-2 (fibroblast growth factor) (PeproTech, Rocky Hill, NJ, USA), 50 ng/mL HGF (hepatocyte growth factor) (PeproTech, Rocky Hill, NJ, USA), 2 µM Y-27632 (Selleckchem Llc., Houston, TX, USA), and 2.5 µM prostaglandin E2 (R&D Systems, Minneapolis, MN, USA) [28]. Cultures were maintained in 5% CO_2_ in a humidified incubator at 37 °C. Medium was replaced every 2–3 days. Small organoid clusters became visible around day 10 (Figure S1A).

### 2.3. Preparation of Organoids and Placental Tissues for IHC-IF

For paraffin embedding, organoids embedded in Matrigel were gently collected into cold medium, washed with PBS containing 0.1% BSA (Carl Roth, Karlsruhe, Germany), fixed in ROTI^®^ Histofix solution (Carl Roth, Karlsruhe, Germany) for 30 min on ice, washed twice with PBS containing 0.1% BSA and stored at least overnight in 70% ethanol at 4 °C. For visualization, organoids were stained with a drop of hematoxylin for 5–10 min, washed gently with Aqua dest., mixed with 100 µL Histowax (Leica Biosytems, Nussloch, Germany), and incubated for 30 min on ice for embedding. Five µm sections were prepared. After deparaffinization, the slides were incubated with the target retrieval solution from DAKO EnVision^TM^ FLEX Kit (DAKO, Hamburg, Germany) for 15 min, or 30 min in the case of placental tissue sections in a water bath (100 °C), blocked with peroxidase for 5 min, and incubated with primary antibodies for 60 min at room temperature. Following primary antibodies were used: rabbit monoclonal p21 (#2947; Cell Signaling Technology, Danvers, MA, USA), mouse monoclonal cytokeratin 7 (#M7018; DAKO, Hamburg, Germany), mouse monoclonal E-cadherin (#610181; BD Transduction Laboratories, San Jose, CA, USA), rabbit monoclonal epidermal growth factor receptor (EGFR) (#4267; Cell Signaling, Technology, Danvers, MA, USA), mouse monoclonal human leukocyte antigen G (HLA-G) (#11-291-C100; Exbio Praha, a.s., Vestec, Czech), and mouse monoclonal Ki67 (#M7240; DAKO, Hamburg, Germany). This was followed by the incubation with secondary antibodies for 30 min at room temperature: goat anti-rabbit or goat anti-mouse Alexa Fluor^®^ 594 (#ab150080 or #ab150116; Abcam, Cambridge, UK) and goat-anti mouse DyLight^®^ 488 or goat anti-rabbit Alexa Fluor^®^ 488 (#ab96879 or #ab150077; Abcam, Cambridge, UK). DAPI (4′,6-diamidino-2-phenylindole-dihydrochloride; Roche, Mannheim, Germany) was used to stain the DNA content. Slides were examined with an AxioObserver.Z1 microscope (Zeiss, Göttingen, Germany) equipped with an AxioCam MRm camera (Zeiss, Göttingen, Germany).

### 2.4. Isolation of Primary Villous Cytotrophoblasts from Human First Trimester Placenta

Isolation of villous cytotrophoblasts from first trimester placenta was performed according to Haider et al. [29] and Vondra et al. [30] using three consecutive digestion steps followed by Percoll density gradient centrifugation (5–70%; Sigma-Aldrich, Taufkirchen, Germany). Placental tissues were washed with PBS and placental villi were minced. The digestion steps were performed in 50 mL tubes at 37 °C in a shaker with 200 rpm for 8 min, 15 min, and 15 min. The digestion buffer (10× HBSS (Hank’s Balanced Salt) [31], 7.5% NaHCO₃, 1 M HEPES (4-(2-hydroxyethyl)-1-piperazineethanesulfonic acid)) was supplemented with 0.25% trypsin (Gibco, Life Technologies, Carlsbad, CA, USA) and 1.25 mg/mL DNase I (Sigma-Aldrich, Taufkirchen, Germany). The second and third digestion steps were pooled and purified with a Percoll gradient as detailed for term placental cytotrophoblasts isolation (see below).

### 2.5. Isolation of Placental Mesenchymal Stem/Stromal Cells from First Trimester Placental Tissue

The isolation protocol by Stiegman et al. [32] was modified. Villous like structures were washed twice, minced, and mixed with 15 mL collagenase II (275 U/mL; Worthington, Columbus, OH, USA), diluted in HBSS, and 500 µL Dispase (90 U/mL; Roche, Mannheim, Germany). After 75 min at 37 °C and 200 rpm, the enzyme activity was stopped with medium containing 10% FBS (Biochrome, Berlin, Germany) and filtered through a 100 μm mesh. Erythrocytes were lysed with lysis buffer and resuspended in DMEM (Gibco, Carlsbad, CA, USA) containing 20% FBS (Biochrome, Berlin, Germany), 1% penicillin/streptomycin, 1 µg/mL amphotericin B (Sigma-Aldrich, Taufkirchen, Germany), and 5 ng/mL hFGF (Promega GmbH, Walldorf, Germany). The medium was changed after 24 h to remove non-adherent cells.

### 2.6. Isolation of Primary Villous Cytotrophoblasts from Human Term Placental Tissue

Villous cytotrophoblast cell isolation and purification was carried out according to Petroff et al. [31]. In brief, approximately 50 g of villous placental tissue free of calcification or hematoma was finely minced within 30 min after delivery, rinsed with 0.9% NaCl, and digested with 0.25% trypsin (Thermo Fisher Scientific, Dreieich, Germany) and 300 U/mL DNase I (Sigma-Aldrich, Taufkirchen, Germany) for 20 min shaking with 200 rpm at 37 °C. After digestion, the supernatant was transferred into tubes containing 1.5 mL FBS (Merck Millipore, Darmstadt, Germany) and centrifuged (1000× *g*, 15 min). The digestion, transfer, and centrifugation steps were repeated two more times. The pellet was resuspended in DMEM (Thermo Fisher Scientific, Dreieich, Germany) and filtered with a 100 μm cell strainer (Corning, NY, USA). The cells were centrifuged (1000× *g*, 10 min), resuspended in Ca/Mg-free Hank’s balanced salt solution, and stratified on two Percoll gradients (5–70%; Sigma-Aldrich, Taufkirchen, Germany). The gradients were centrifuged without brake (1200× *g*, 20 min). The fractions between 35 and 50% of the gradients were used, pooled, and diluted in pre-warmed medium for centrifugation (1000× *g*, 5 min). The cell pellet was resuspended in erythrocyte lysis buffer (155 mM NH_4_Cl, 10 mM KHCO_3_, and 0.1 mM EDTA) [27], incubated for 10 min at 37 °C, centrifuged (1000× *g*, 5 min), and washed twice with PBS. The remaining cells were seeded onto 15 cm cell culture plates in DMEM/F12 (Life Technologies) containing 20% FBS (Biowest, Riverside, CA, USA), 100 μg/mL streptomycin, 100 U/mL penicillin, and 1 µg/mL amphotericin B (Sigma-Aldrich, Taufkirchen, Germany) for 45 min to remove adherent stromal cells. Non-adherent trophoblastic cells were collected, seeded onto collagen-coated plates (Greiner Bio-One, Frickenhausen, Germany), and cultured under standard cell culture conditions.

### 2.7. Immunohistochemistry of Placental Tissue

A standard staining procedure with DAKO EnVision^TM^FLEX Kit (#K8000; DAKO, Hamburg, Germany) was used to stain FFPE placental tissue sections from PE patients and matched controls, as stated [33]. The following antibodies were used: mouse monoclonal antibody against p21 (#2946; 1:25, incubation 1 h at 37 °C), rabbit monoclonal antibody against p27 (#3686; 1:30, incubation 1 h), rabbit polyclonal antibody against p57 (#2557; 1:30, incubation 1 h; Cell Signaling Technology, Danvers, MA, USA), and p-p21 (Thr-145, #AF3290; 1:30, incubation 1 h at 37 °C; Affinity Biosciences, Cincinnati, OH, USA). Slides were counterstained with hematoxylin and analyzed using an AxioObserver.Z1 microscope (Zeiss, Göttingen, Germany). Negative controls included samples stained with control immunoglobulin G (IgG) lacking primary antibody. Evaluation was carried out without knowing the diagnosis. The average of the percentage of positive cells was determined. In the case of syncytiotrophoblast (STB), the positive area per visual field was estimated. Ten fields per sample were counted. The slides were further evaluated by the semi-quantitative H-score method, which takes the staining intensity into account. The H-score is determined by adding the results of multiplying the percentage of positive stained cells with their staining intensity (scored as 0 for no signal, 1 = weak, 2 = moderate and 3 = strong): [1 × (% cells 1) + 2 × (% cells 2) + 3 × (% cells 3)]. The highest possible value is 300 [34,35].

### 2.8. RNA Extraction and Real-Time Quantitative PCR

Total RNAs were extracted with EXTRACTME Total RNA Kit, with DNase digestion for tissue samples or without DNase digestion for cell lines, according to manual instructions (7Bioscience GmbH, Neuenburg, Rhein, Germany). In the case of first trimester samples, total RNAs were extracted from FFPE tissues using the ReliaPrep™ FFPE Total RNA Miniprep System as instructed (Promega GmbH, Walldorf, Germany). Reverse transcription was performed using the Go Script Reverse Transcription Mix as instructed (Promega GmbH, Walldorf, Germany). A StepOnePlus Real-time PCR System (Applied Biosystems, Darmstadt, Germany) was used to perform real-time quantitative PCR and data were analyzed with StepOne Software v2.3 (Applied Biosystems, Darmstadt, Germany). To analyze primary placental tissue, the mean value of expression levels of *SDHA* (succinate dehydrogenase complex, subunit A), *TBP* (TATA box-binding protein), and *YWHAZ* (tyrosine 3-monooxygenase/tryptophan 5-monooygenase activation protein, zeta polypeptide) or *TBP* alone served as the endogenous control [36,37]. For gene evaluation from cultured cells *GAPDH* (glyceraldehyde 3-phosphate dehydrogenase) was used as the endogenous control. The primers and probes for *GAPDH* (Hs_02786624), *CDKN1A* (Hs00355782_m1), *CDKN1B* (Hs_00153277_m1), *CDKN1C* (Hs00175938_m1), *TP53* (Hs01034249_m1), *HERV-FRD* (Hs01942443_s1), *CGbeta5* (Hs00361224_gH), *GCM1* (Hs_00961601_m1), *GATA3* (Hs00231122_m1), *TFAP2A* (Hs01029413_m1), *TFAP2C* (Hs00231476_m1)*, ELF5* (Hs01063023_g1), *SDHA* (Hs00188166_m1), *TBP* (Hs99999910_m1), and *YWHAZ* (Hs00237047_m1) were obtained from Applied Biosystems (Darmstadt, Germany). All results were shown as relative quantification (RQ) [38].

### 2.9. Cell Culture, Transfection and Treatment

The HTR-8/SVneo cell line (referred to as HTR) was kindly provided by Prof. Dr. Charles Graham [39] and the SGHPL-4 cell line by Prof. Dr. Guy Whitley [40]. BeWo (Sigma-Aldrich, Taufkirchen, Germany) and JEG-3 cells (ATCC, Wesel, Germany) were cultured as instructed. The 1% oxygen atmosphere was supplied in a special hypoxic O_2_ incubator (Galaxy 48 R, Eppendorf, Hamburg, Germany).

siRNA targeting p21 (sense: ACACCUCCUCAUGUACAUA and antisense: UAUGUACAUGAGGAGGUGU; designated as sip21) was manufactured by Sigma-Aldrich (Taufkirchen, Germany). A different siRNA against p21 (referred to as sip21 #2), containing a mixed pool of siRNAs, was obtained from Santa Cruz (Heidelberg, Germany; sc-29427). Control siRNA was obtained from Qiagen (Hilden, Germany; #1027281). siRNAs (30 nM). Cells were transiently transfected with siRNA with Oligofectamine^TM^ (Thermo Fisher Scientific, Dreieich, Germany) [41].

Trophoblast fusion was induced by treating cells with 25 μM forskolin (Sigma-Aldrich, Taufkirchen, Germany) for indicated time points. An equal amount of dimethyl sulfoxide (DMSO, Sigma-Aldrich, Taufkirchen, Germany) was used as the vehicle control.

### 2.10. Western Blot Analysis

Western blot analysis was performed as reported [42]. Cells were harvested with RIPA buffer (50 mM Tris pH 8.0, 150 mM NaCl, 1% NP-40, 0.5% Na-desoxycholate, 0.1% SDS, 1 mM NaF, phosphatase and protease inhibitor cocktail tablets (Roche, Mannheim, Germany)). The following antibodies were used: mouse monoclonal antibody against GAPDH (GTX627408; GeneTex, Eching, Germany) and p53 (DO-1, sc-126; Santa Cruz, Heidelberg, Germany); rabbit monoclonal antibody against p21 (#2947) and p27 (#3686); and rabbit polyclonal antibody against p57 (#2557; Cell Signaling Technology, Danvers, MA, USA). ImageJ 1.48v software (National Institutes of Health, Bethesda, MD, USA) was used for densitometric measurements of western blot analysis.

### 2.11. Immunofluorescence Staining

Indirect immunofluorescence was performed as reported [25,41]. Following primary antibodies were used: rabbit monoclonal antibody against epidermal growth factor receptor (EGFR) (#4267; Cell Signaling, Technology, Danvers, MA, USA), mouse monoclonal antibody against E-cadherin (#610181; BD Transduction Laboratories, San Jose, CA, USA), rabbit polyclonal antibody against human chorionic gonadotropin (β-hCG) (#SAB4500168; Sigma-Aldrich, Taufkirchen, Germany), mouse monoclonal antibody against cytokeratin 7 (#M7018; DAKO, Hamburg, Germany), rabbit monoclonal antibody against cytokeratin 18 (#ab32118; Abcam, Cambridge, UK), mouse monoclonal antibody against human leukocyte antigen G (HLA-G) (#11-291-C100; Exbio Praha, a.s., Vestec, Czech), and mouse monoclonal antibody against vimentin (#M7020; DAKO, Hamburg, Germany). FITC- and Cy3-conjugated secondary antibodies were obtained from Jackson Immunoresearch (Cambridgeshire, UK). DAPI (Roche, Mannheim, Germany) was used to stain the DNA content. Slides were examined with an AxioObserver.Z1 microscope (Zeiss, Göttingen, Germany) equipped with an AxioCam MRm camera (Zeiss, Göttingen, Germany).

### 2.12. Luciferase Assay

BeWo or JEG-3 cells were treated with scrambled siRNA (sicon) or siRNA against the 3′-untranslated region (UTR) of p21 (sip21) or mixed siRNAs against the coding region of p21 (sip21 #2). After 24 h, the syncytin-2 (2 µg) promoter plasmid [43] was transfected with FuGENE^®^ HD transfection reagent (Promega GmbH, Walldorf, Germany) for 48 h. Cells were harvested with cell culture lysis reagent from Promega GmbH (Walldorf, Germany; #E1531) and the assays were performed with the luciferase assay system from Promega GmbH (Walldorf, Germany; #E1501).

### 2.13. Microarray Analysis

BeWo cells were treated with control siRNA or siRNA targeting the UTR of p21 (sip21) for 48 h. Cells from three independent experiments were harvested and the total RNA was isolated using RNeasy Kits (Qiagen, Hilden, Germany). The expression was assessed using Human HT-12 v4 Beadchip (Illumina, San Diego, CA, USA), a direct hybridization whole-gene expression array. The expression profiling service from the German Cancer Research Center (DKFZ Microarray Core Facility, Heidelberg, Germany) was used. The most significant genes with a *p*-value (Student’s *t*-test) less than 0.05 were selected. A full gene list is available as Supplementary Table S1, which shows data from the whole gene-expression array with a *p*-value smaller than 0.05.

### 2.14. Statistical Analysis

Outliers were detected with Grubbs’ test (GraphPath QuickCalcs, San Diego, CA, USA). Data distribution normality was analyzed with the Shapiro–Wilk test and statistical significance was analyzed with the Student’s *t* test, or, if not Gaussian distributed, with the non-parametric Wilcoxon-test (paired) or the Mann–Whitney U test (unpaired samples). Difference was defined as statistically significant when *p* < 0.05.

## 3. Results

### 3.1. Cell Cycle Regulators Are Affected by the Delivery Mode, and Specifically Expressed in Trophoblast Organoids and Placental Tissues

**Figure 1:**
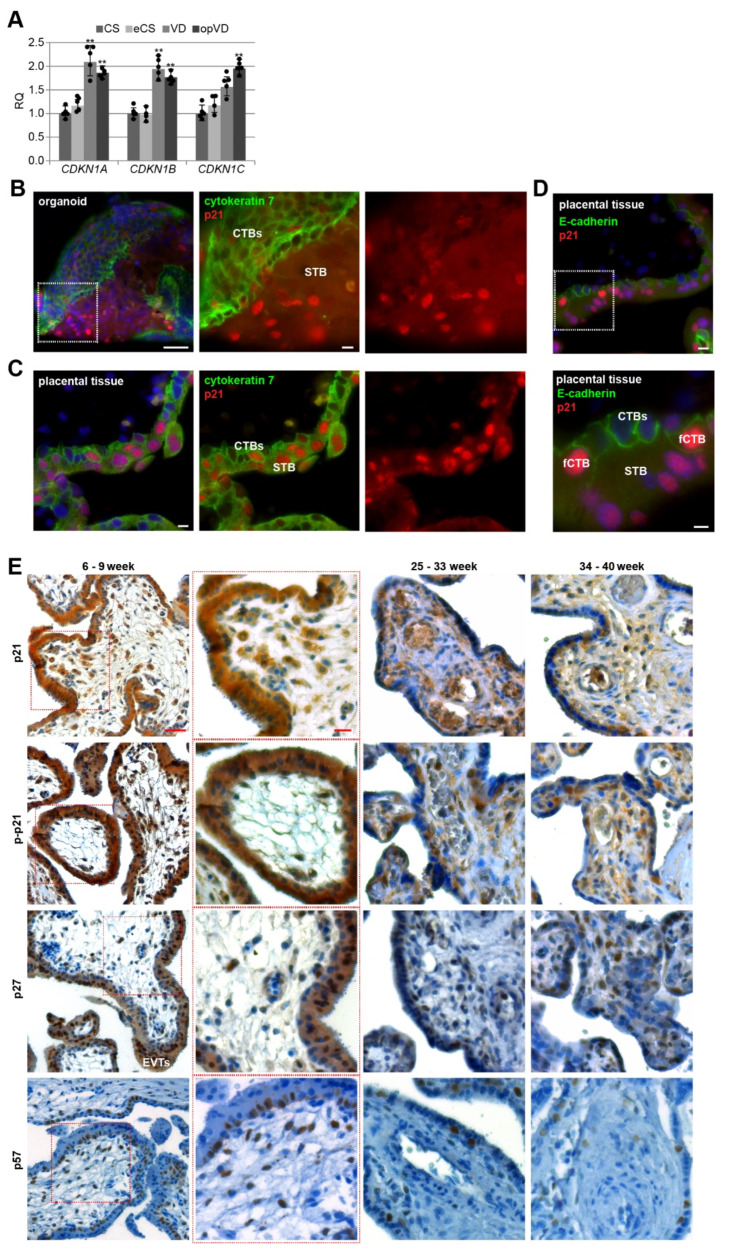
Cell cycle regulators are affected by the delivery mode, and expressed in trophoblast organoids and placental tissues. (**A**) The relative amount of the gene levels of *CDKN1A* (p21), *CDKN1B* (p27), and *CDKN1C* (p57) was analyzed in placental tissues from different delivery modes (*n* = 5). The results are presented as relative quantification (RQ) with minimum and maximum range. The mean value of the expression levels of *SDHA* (succinate dehydrogenase complex, subunit A), *TBP* (TATA box–binding protein), and *YWHAZ* (tyrosine 3–monooxygenase/tryptophan 5–monooxygenase activation protein, zeta polypeptide) served as the endogenous control. CS, caesarean section; eCS, emergency caesarean section after the onset of labor; VD, vaginal delivery; opVD, operative vaginal delivery. Unpaired Student’s *t*-test was used for statistical analysis, ** *p* < 0.01. (**B**) Representative immunohistochemistry-immunofluorescence (IHC–IF) image of a trophoblast organoid (8 weeks of gestation) stained for cytokeratin 7 (green), p21 (red), and nuclei (DAPI, blue) is shown. Scale: 50 µm. Inset scale: 10 µm. Villous cytotrophoblasts (CTBs) and the syncytiotrophoblast (STB) are indicated. (**C**) Representative IHC-IF image of placental tissue (8 weeks of gestation) stained for cytokeratin 7 (green), p21 (red), and nuclei (DAPI, blue) is presented. Scale: 10 µm. (**D**) Representative IHC–IF image of placental tissue (7 weeks of gestation) stained for E-cadherin (green), p21 (red), and nuclei (DAPI, blue) is shown. Scale: 10 µm. Inset scale: 5 µm. CTBs ongoing to fuse to the STB (fCTBs), CTBs and the STB are indicated. (**E**) Formalin-fixed and paraffin-embedded (FFPE) tissue sections were immunohistochemically stained with p21 (first row), p–p21 (second row), p27 (third row) or p57 (fourth row) antibody (brown), respectively, and counterstained with hematoxylin (blue). Scale: 50 µm. Inset scale: 20 µm. EVTs (extravillous cytotrophoblasts) are indicated.

The rapid expansion of the human placenta is attributed to spatiotemporally regulated cell proliferation, for which the cell cycle regulators are indispensable. A previous study showed that the mode of delivery affected the expression of certain genes including *CDKN1C* (p57), which was elevated over 2-fold in placental tissues from labor deliveries compared to elective caesarean sections [44]. We started to verify and extend this very important issue in terms of the impact of the delivery mode on gene expression of *CDKN1A* (p21), *CDKN1B* (p27), and *CDKN1C* (p57). Placental tissues were collected from healthy women with different delivery modes without significant differences in gestational age, maternal age, and maternal body mass index (BMI) (Table 1). Total RNAs were extracted for gene analysis. We compared the gene expression levels of placental tissues from elective caesarean section (CS), emergency caesarean section after the onset of labor (eCS), vaginal delivery (VD), and operative vaginal delivery (opVD, mainly forceps delivery). Indeed, as shown in Figure 1A, the delivery mode strongly affected the gene expression of cell cycle regulators in the human placenta, especially for *CDKN1A* and *CDKN1B*, where a significant increase of about 2-fold was obtained after VD and opVD delivery. For *CDKN1C*, a significant 2-fold elevation was observed by opVD. Based on these data, we decided to collect placental tissues from caesarean sections without mechanical compression caused by uterine contractions or additional stress factors.

To gain insight into the possible roles of cell cycle regulators in placental development, we generated human trophoblast 3D organoids (ORGs) according to well-established protocols [26,28,29]. Villous cytotrophoblasts (CTBs) were purified from first trimester placental tissues and embedded in Matrigel or plated on ultra-low attachment plates (Figure S1A). The trophoblast origin was corroborated by immunohistochemistry-immunofluorescence (IHC-IF) staining of the presence of the trophoblast marker cytokeratin 7 and epidermal growth factor receptor (EGFR), the proliferation marker Ki67, and the absence of human leukocyte antigen G (HLA-G) (Figure S1B), and by analyzing the gene expression of *GATA3* (GATA binding protein 3), *TFAP2A* (transcription factor AP-2 alpha), *TFAP2C* (transcription factor AP-2 gamma), and *ELF5* (E74 like ETS transcription factor 5) (Figure S1C), previously described trophoblast identity criteria [28]. The data revealed similar gene expression profiles between first trimester organoids (ORGs) and isolated primary cytotrophoblasts (pCTBs); both displayed higher expression levels of *GATA3*, *TFAP2A*, *TFAP2C,* and *ELF5* compared to placental mesenchymal stem/stromal cells (pMSCs) isolated from first trimester placental tissues. Additionally, relative to first trimester pCTBs, trophoblast organoids expressed very high levels of *CGbeta5* (β-hCG) (Figure S1D). IHC-IF staining for cytokeratin 7 revealed that organoids had the previously described inside-out structure [28,29], where the CTBs formed the outer layer and fused toward the center to generate the STB (Figure 1B and Figure S1B). In comparison, the IHC-IF of first trimester placenta showed the familiar structure (Figure 1C). Interestingly, in both trophoblast organoids and placental tissue sections, p21 was expressed in CTBs and the STB (Figure 1B,C), in the nucleus and the cytoplasm.

For further analyses, we used sections of placental tissues with gestational ages between six and nine weeks, between 25 and 33 weeks as well as between 34 and 40 weeks from normal donors. Using IHC, placental sections were stained for p21, p–p21 (Thr-145), p27 or p57, and counterstained with hematoxylin. Whereas p21’s role in cell cycle arrest is attributed to its nuclear localization, its phosphorylation at Thr-145 by distinct kinases is described as a marker for cytoplasmic translocation or enhanced protein stability contributing to cell cycle progression [23]. Placental tissue was highly positive for p21, p–p21, and p27 (Figure 1E). The positive staining was found in the cytoplasm as well as the nucleus of trophoblastic cells of the placenta, especially in the proliferative villous CTBs, in particular, CTBs ongoing to fuse to the STB, so called fCTBs marked by partial loss of E-cadherin staining, suggesting the breakdown of apical and lateral plasma membranes (fCTBs, Figure 1D), the terminally differentiated, non-proliferative, and multinucleated STB, the migrating EVTs in proliferative cell columns, and villous stromal cells throughout gestation. The positive staining of p57 was predominantly present in the nucleus of fCTBs, stromal cells, and EVTs (Figure 1E, last panel).

### 3.2. Cell Cycle Regulators Are Highly Expressed during the First Trimester of Gestation

**Figure 2:**
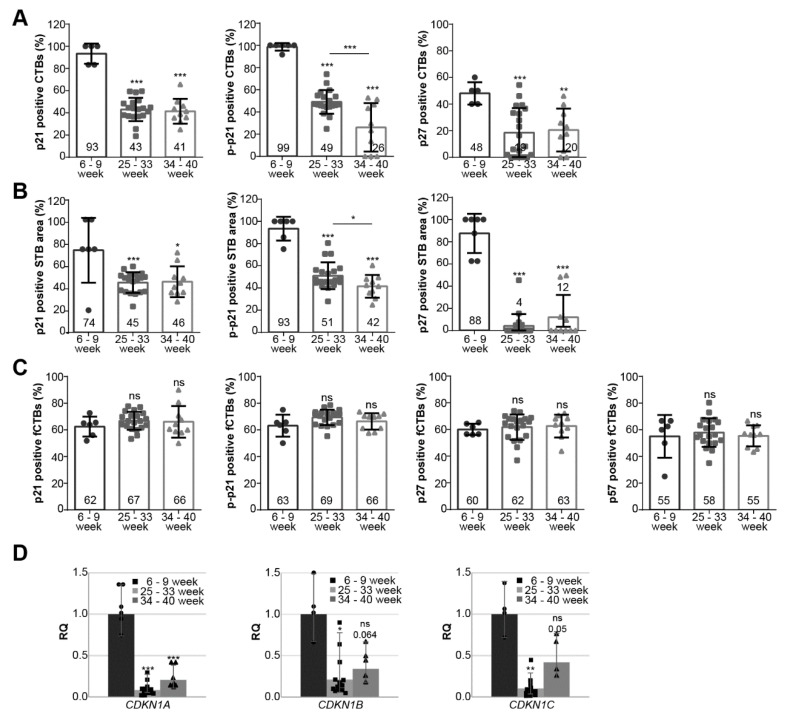
Cell cycle regulators are highly expressed during the first trimester of gestation. (**A**–**C**) Evaluation of positive cells in first trimester placental sections (*n* = 6; named 6–9 week), early-onset control (*n* = 20; named 25–33 week), and late-onset control samples (*n* = 10; named 34–40 week). The results are presented as bar and scatter plots showing the mean value ± SD. (**A**) Quantification of p21 (left panel), p–p21 (middle panel), and p27 (right panel) positive CTBs in %. (**B**) Quantification of p21 (left panel), p-p21 (middle panel), and p27 (right panel) positive stained STB area in %. (**C**) Quantification of p21 (left panel), p–p21 (second panel), p27 (third panel), and p57 (right panel) positive fCTBs in %. (**D**) The relative amount of the gene levels was analyzed from placental tissues: left panel *CDKN1A* (p21), middle panel *CDKN1B* (p27), and right panel *CDKN1C* (p57). The results are presented as relative quantification (RQ) with minimum and maximum range. *TBP* was used as the endogenous control. Unpaired Student’s *t*-test or Mann–Whitney U test referring to first trimester samples was used for statistical analysis, * *p* < 0.05, ** *p* < 0.01, *** *p* < 0.001. CTBs, cytotrophoblasts; fCTBs, cytotrophoblasts ongoing to fuse; STB, syncytiotrophoblast.

We analyzed the positively stained CTBs, fCTBs, and the STB area for p21, p–p21, p27, and p57 in tissue sections from healthy donors with gestational ages between six and nine weeks (*n* = 6), between 25 and 33 weeks (*n* = 20; Table 2, also served as early-onset control group), and between 34 and 40 weeks (*n* = 10; Table 3, also served as late-onset control group) (representatives are shown in Figure 1E). In the first trimester sections, there were not enough EVTs for a reliable quantification. For all staining, first trimester sections showed the highest percentage of positive CTBs and positive STB area with a significant decline in the early- and late-onset control group (Figure 2A,B; named 25–33 or 34–40 week). There was a significant difference in p–p21 positive staining of CTBs and the STB area between early- and late-onset control samples (Figure 2A,B, middle panel). Interestingly, fCTBs displayed high percentages of positive staining of p21, p–p21, p27, and p57 throughout gestation (Figure 2C), suggesting that this cell population is highly active in proliferation and differentiation. Next, the gene expression of *CDKN1A* (p21), *CDKN1B* (p27), and *CDKN1C* (p57) was evaluated in placental tissues from the first trimester samples (named 6–9 week) compared to early- and late-onset controls (Figure 2D). The relative amounts of *CDKN1A*, *CDKN1B,* and *CDKN1C* in early-onset controls were significantly reduced by 92%, 79%, and 90%, respectively, compared to the first trimester group. Particularly, *CDKN1A* was significantly reduced in early- as well as late-onset controls compared to the first trimester samples (Figure 2D, left). In addition, a moderate increase was observed in the gene expression of all cell cycle regulators in late-onset controls compared to early-onset controls (Figure 2D).

### 3.3. p21 Expression Is Reduced in fCTBs of Early-Onset PE Placental Samples

**Figure 3:**
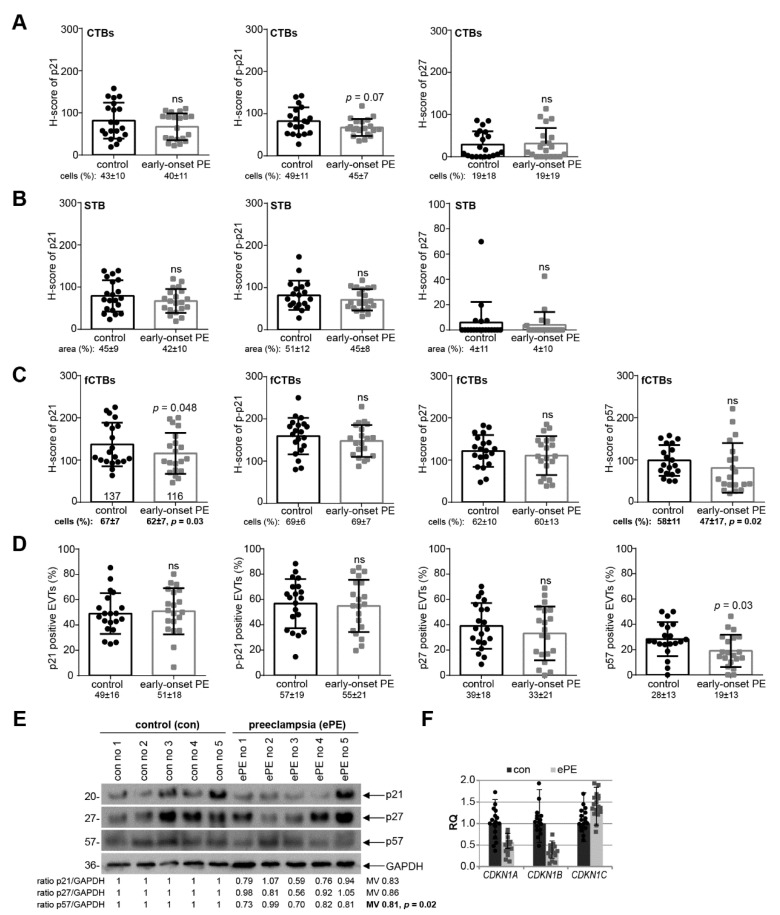
p21 expression is reduced in fCTBs of early-onset PE samples. (**A**–**C**) Quantification of cell cycle regulators in placental sections of control donors (control, *n* = 20) and patients with early-onset PE (ePE, *n* = 20) using the H-score method. The results are presented as bar and scatter plots showing the mean value with SD. The percentage of positive stained cells/area is shown under each graph. (**A**) H-score of p21 (left panel), p–p21 (middle panel), and p27 (right panel) for CTBs. (**B**) H-score of p21 (left panel), p–p21 (middle panel), and p27 (right panel) for the STB area. (**C**) H-score of p21 (left panel), p–p21 (second panel), p27 (third panel), and p57 (right panel) for fCTBs. (**D**) Quantification of p21 positive (left panel), p–p21 positive (second panel), p27 positive (third panel), and p57 positive EVTs (right panel) in %. (**E**) Western blot analysis with extracts from placental tissues is shown. Glyceraldehyde-3-phosphate dehydrogenase (GAPDH) served as the loading control. (**F**) The relative amount of the gene levels of *CDKN1A* (p21), *CDKN1B* (p27), and *CDKN1C* (p57) was analyzed from placental tissues. The results are presented as relative quantification (RQ) with minimum and maximum range. *TBP* was used as the endogenous control. Paired Student’s *t*-test or Wilcoxon-test was used for statistical analysis. CTBs, cytotrophoblasts; fCTBs, cytotrophoblasts ongoing to fuse; STB, syncytiotrophoblast; EVT, extravillous cytotrophoblasts; no, number; MV, mean value.

To address if cell cycle regulators were altered in preeclamptic placentas, the expression levels of p21, p–p21, p27, or p57 were compared between twenty early-onset PE placental tissues and twenty samples from well-matched control donors (Table 2) by the semi-quantitative H-score method, which combines the percentage of stained cells/area and their staining intensity. There was no apparent difference in cell cycle regulators in the H-score of CTBs or the STB, and the percentage of positive CTBs or in the positive stained area per visual field in the STB in early-onset PE samples compared to their respective control counterparts (Figure 3A,B). Interestingly, a significant reduction of p21 in the H-score of fCTBs as well as in the percentage of positive fCTBs were observed in early-onset preeclamptic placental samples, in comparison to the matched control tissues (Figure 3C, left graph). While p–p21 and p27 were almost comparable (Figure 3C, 2nd and 3rd graph), the percentage of positive p57 fCTBs declined in early-onset PE samples compared to the control tissues (Figure 3C, right graph, bottom). Moreover, the percentage of p57 positive stained EVTs was significantly reduced, whereas p21, p–p21, and p27 were hardly altered (Figure 3D). To underscore these results, we next evaluated the expression with western blot analyses from whole tissue samples. While the expression of p21, p27, and p57 was decreased, only p57 showed a significant reduction (Figure 3E). A limitation of our study is that the p–p21 antibody did not work for tissue western blot analysis. Further gene analysis showed reduced levels of *CDKN1A* (p21) and *CDKN1B* (p27) (Figure 3F).

Ten late-onset PE samples and ten control tissues from well-matched donors (Table 3) were also systematically analyzed. Obvious change in p21, p–p21, p27, and p57 expression was not observed at protein as well as at gene level (Figure S2). Interestingly, the H-score of p–p21 was significantly elevated in the STB (Figure S2B, middle graph). In sum, these data support the notion that early- and late-onset PE derive from distinct pathogenesis and should be considered separately from each other.

### 3.4. Reduced p21 Protein Expression in Early-Onset PE with HELLP Syndrome

**Figure 4:**
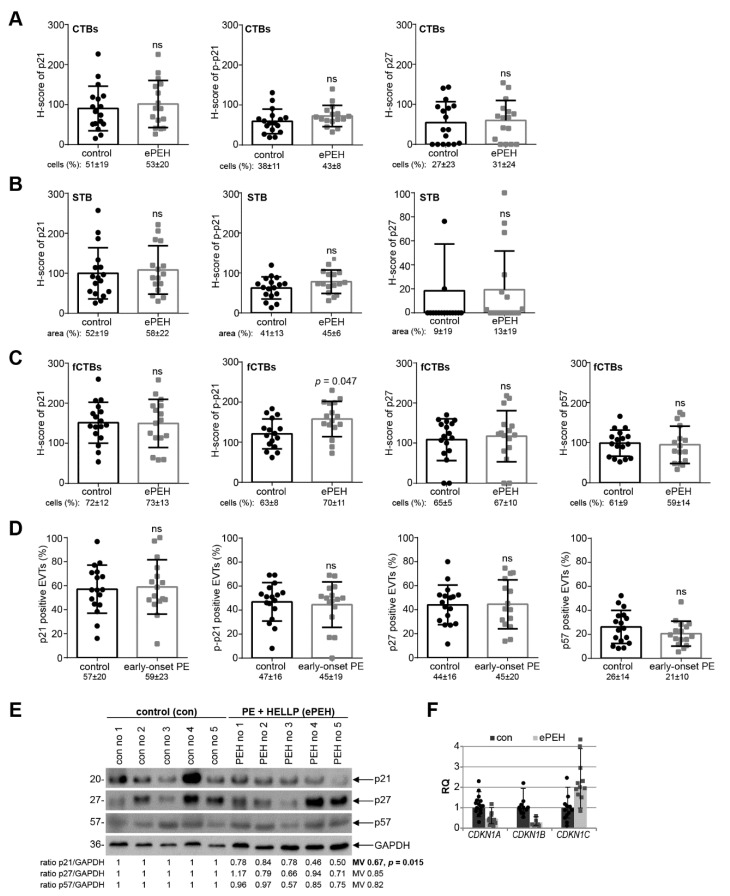
p21 expression is reduced in early-onset PE complicated by the HELLP syndrome. (**A**–**C**) Quantification of cell cycle regulators in placental sections of control donors (control, *n* = 17) and placental tissues from early-onset PE complicated by the HELLP syndrome (ePEH, *n* = 16) using the H-score method. The results are presented as bar and scatter plots showing the mean value with SD. The percentage of positive stained cells/area is shown under each graph. (**A**) H-score of p21 (left panel), p–p21 (middle panel), and p27 (right panel) for CTBs. (**B**) H-score of p21 (left panel), p–p21 (middle panel), and p27 (right panel) for the STB area. (**C**) H-score of p21 (left panel), p–p21 (second panel), p27 (third panel), and p57 (right panel) for fCTBs. (**D**) Quantification of p21 positive (left panel), p–p21 positive (second panel), p27 positive (third panel), and p57 positive EVTs (right panel) in %. (**E**) Western blot analysis with extracts from placental tissues is shown. GAPDH served as the loading control. (**F**) The relative amount of the gene levels of *CDKN1A* (p21), *CDKN1B* (p27) and *CDKN1C* (p57) was analyzed with placental tissues. The results are presented as relative quantification (RQ) with minimum and maximum range. *TBP* was used as the endogenous control. Paired Student’s *t*-test or Wilcoxon-test was used for statistical analysis. CTBs, cytotrophoblasts; fCTBs, cytotrophoblasts ongoing to fuse; STB, syncytiotrophoblast; EVT, extravillous cytotrophoblasts; no, number; MV, mean value.

Since early-onset PE is often complicated by the HELLP syndrome (ePEH) [1], placental samples were also collected from patients with ePEH and their well-matched controls (Table 4). The percentage of positive cells/area and the staining intensity of CTBs, the STB, and fCTBs were evaluated for p21, p–p21, p27, and p57 (Figure 4A–C). There was no obvious difference of cell cycle regulators in the H-score of CTBs or the STB, and the percentage of positive CTBs or in the positive stained area of the STB in ePEH compared to the well-matched controls (Figure 4A,B). Interestingly, there was a significant increase in the H-score of p–p21 in fCTBs (Figure 4C). The percentage of positive stained EVTs was comparable (Figure 4D). Western blot analyses with cellular lysates from whole tissue samples showed that the expression of p21, p27, and p57 was decreased, and only p21 showed a significant reduction (Figure 4E). Further gene analysis showed reduced levels of *CDKN1A* (p21) and *CDKN1B* (p27) (Figure 4F), as observed in early-onset PE (Figure 3F).

### 3.5. p21 Expression Is Decreased in Trophoblastic Cell Lines and in Isolated Primary Cytotrophoblasts under Hypoxic Conditions

**Figure 5:**
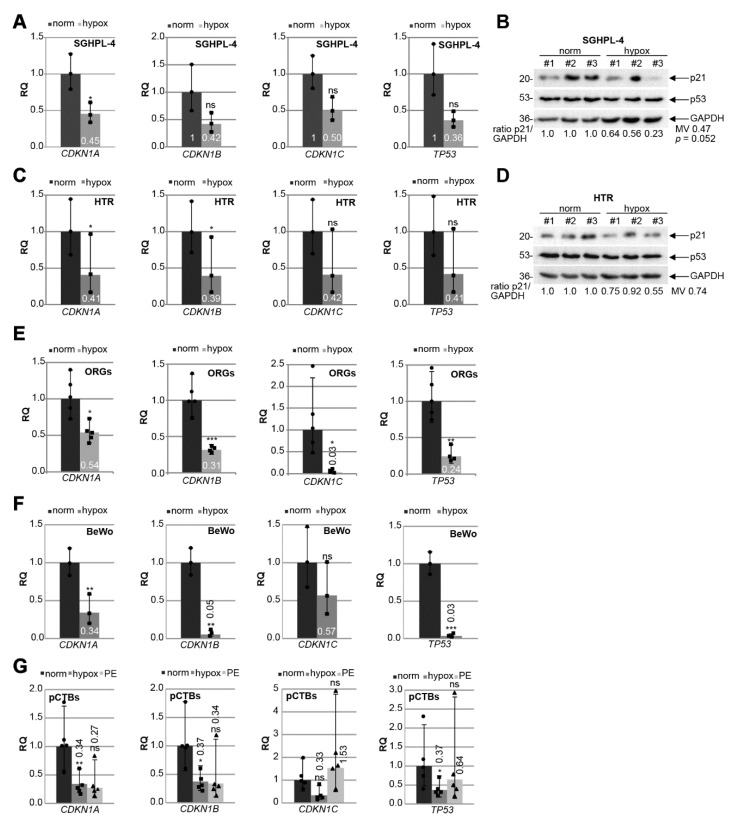
p21 expression is decreased in trophoblastic cell lines and in isolated primary trophoblasts under hypoxic conditions. Cells were grown under normoxia (norm, 21.4% O_2_) or hypoxia (hypox, 1% O_2_) for 48 h prior to RNA extraction or western blot analysis. (**A**) SGHPL-4 cells. Gene analysis of *CDKN1A* (p21), *CDKN1B* (p27), *CDKN1C* (p57) and *TP53* (p53) is shown. The results (*n* = 3) are presented as relative quantification (RQ) with minimum and maximum range. *GAPDH* was used as the endogenous control. (**B**) Western blot analysis with cellular lysates from SGHPL-4 cells (*n* = 3). GAPDH served as the loading control. (**C**) HTR cells. Gene analysis of *CDKN1A* (p21), *CDKN1B* (p27), *CDKN1C* (p57), and *TP53* (p53) is shown. The results (*n* = 3) are presented as relative quantification (RQ) with minimum and maximum range. *GAPDH* was used as the endogenous control. (**D**) Western blot analysis with cellular lysates from HTR cells (*n* = 3). GAPDH served as the loading control. (**E**) Organoids (ORGs) cultured under normoxia (norm; *n* = 5) or hypoxia (hypox; *n* = 4). Relative gene levels of *CDKN1A* (p21), *CDKN1B* (p27), *CDKN1C* (p57), and *TP53* (p53) are shown. The results are presented as relative quantification (RQ) with minimum and maximum range. *GAPDH* was used as endogenous control. (**F**) BeWo cells. Relative gene levels of *CDKN1A* (p21), *CDKN1B* (p27), *CDKN1C* (p57), and *TP53* (p53) are shown. The results (*n* = 3) are presented as the relative quantification (RQ) with minimum and maximum range. *GAPDH* was used as the endogenous control. (**G**) Primary trophoblasts (pCTB) were isolated from healthy and preeclamptic (PE) donors. pCTBs from healthy donors were cultured under normoxia (norm; dot) or hypoxia (hypox, square), PE (triangle) pCTBs were grown under normoxia. The gene level of *CDKN1A* (p21), *CDKN1B* (p27), *CDKN1C* (p57), and *TP53* (p53) was evaluated (*n* = 5). *GAPDH* was used as the housekeeping gene control. Student’s *t*-test, * *p* < 0.05, ** *p* < 0.01, *** *p* < 0.001. ns, not significant; MV, mean value.

PE is associated with chronic hypoxia of the placenta through defective trophoblast invasion and inadequate remodeling of the maternal spiral arteries [5,17,45,46]. To mimic the situation in PE, immortalized first trimester trophoblast cell lines SGHPL-4 [40] and HTR [39] were grown under normal (21.4% O_2_) or hypoxic conditions (1% O_2_) for 48 h. The gene levels of *CDKN1A* (p21), *CDKN1B* (p27), *CDKN1C* (p57), and *TP53* (p53), the master regulator of p21, were measured. *CDKN1A* was significantly reduced under hypoxic conditions in SGHPL-4 cells, whereas *CDKN1B*, *CDKN1C,* and *TP53* were decreased but not significantly (Figure 5A). Moreover, the p21 protein was also reduced, while the p53 protein expression was not affected by hypoxia (Figure 5B). Comparable results were also observed with HTR cells, which showed a significantly lowered gene expression of *CDKN1A* and *CDKN1B* (Figure 5C) as well as decreased p21 protein expression under hypoxic conditions (Figure 5D). To investigate cell cycle regulators in a more physiological setting, trophoblast organoids (ORGs) derived from first trimester placenta were generated and cultured under normal (21.4% O_2_) or under hypoxic conditions (1% O_2_) for 48 h. The expression of cell cycle regulator genes was also affected, showing a significant decline in *CDKN1A*, *CDKN1B*, *CDKN1C*, and *TP53* under hypoxia (Figure 5E). The choriocarcinoma cell line BeWo, a widely used cell culture model mimicking CTB fusion and differentiation [47], was also grown under normoxia and hypoxia for gene analysis. *CDKN1A*, *CDKN1B*, and *TP53* were significantly reduced under low oxygen supply (Figure 5F).

To further underline the observations, human primary cytotrophoblasts (pCTBs) were isolated from five term placentas of healthy donors, which were grown under normoxia or hypoxia and compared to pCTBs isolated from late-onset PE patients grown under normoxic conditions (Table 5). The characterization of isolated trophoblastic cells was performed with positive staining of EGFR, E-cadherin, β-hCG, cytokeratin 7, cytokeratin 18, and negative markers HLA-G and vimentin (Figure S3). Compared to normoxia, the gene levels of *CDKN1A* (p21), *CDKN1B* (p27), and *TP53* (p53) were significantly reduced in primary cytotrophoblasts under hypoxic conditions (Figure 5G). Interestingly, a decrease in *CDKN1A* and *CDKN1B* expression was also observed in the pCTBs from late-onset PE placentas, albeit not significant due to the small sample size (Figure 5G).

### 3.6. Knockdown of p21 Impairs the Fusion Ability of Trophoblastic BeWo Cells

**Figure 6:**
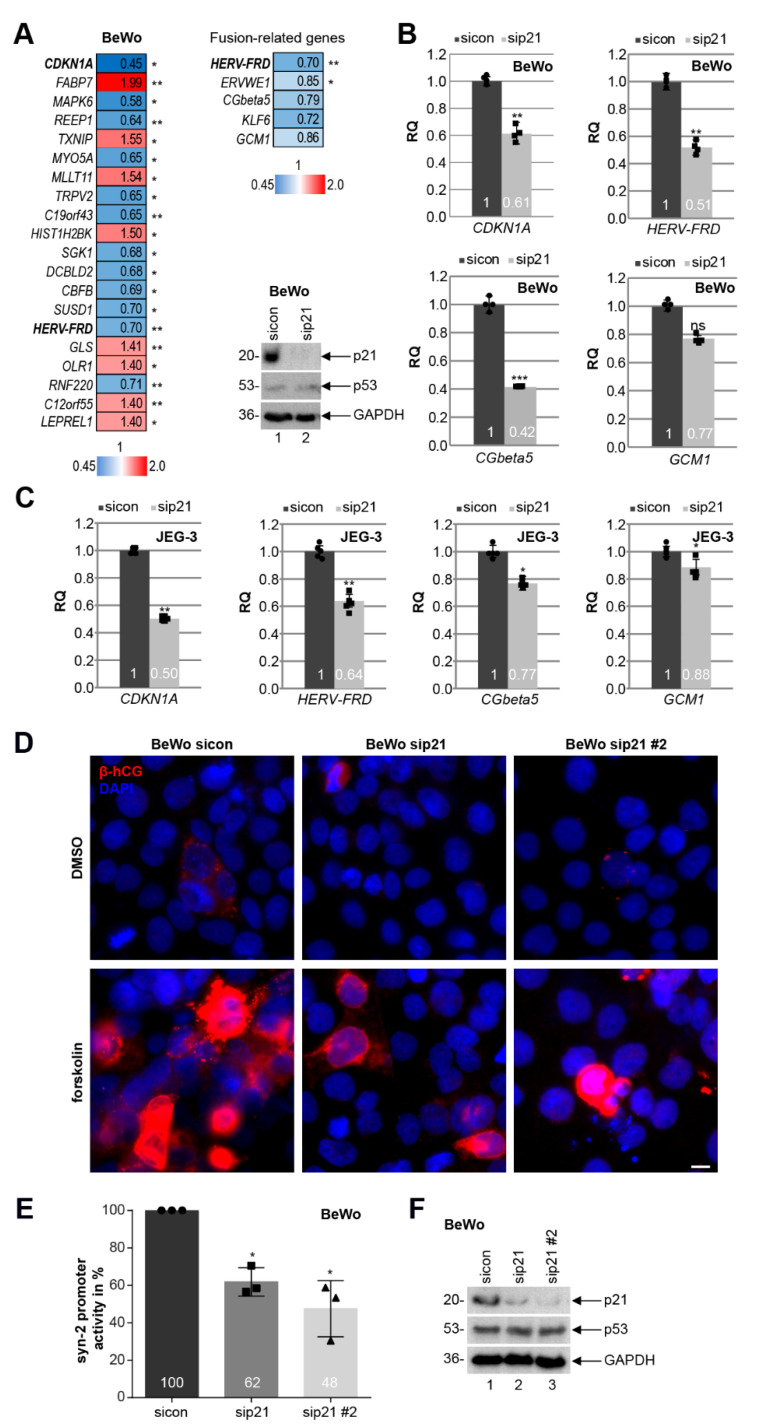
Knockdown of p21 impairs the fusion ability of BeWo and JEG-3 cells. (**A**) Left panel: genome-wide profiling of BeWo cells treated with sicon or sip21 for 48 h. Total RNAs were extracted from three independent experiments. Gene expression was analyzed using HumanHT-12 v4 beadchip array. Genes with a *p*-value < 0.05 are included. Heatmap of the most differently expressed genes are shown (greater than 1, red color code; below 1, blue color code). Right upper panel: heatmap of fusion-related genes is depicted. Right lower panel: western blot analysis was performed with cellular lysates from BeWo cells treated with scrambled siRNA (sicon) or siRNA targeting the untranslated region (UTR) of p21 (sip21). GAPDH was taken as the loading control. (**B**) Gene analysis of BeWo cells depleted of p21. The mRNA levels of p21 (*CDKN1A*), syncytin-2 (*HERV-FRD*), β-hCG (*CGbeta5*), and *GCM1* are shown. *GAPDH* was used as the housekeeping gene control. The results are presented as RQ with minimum and maximum range (*n* = 4). (**C**) Gene analysis of JEG-3 cells depleted of p21. The mRNA levels of p21 (*CDKN1A*), syncytin-2 (*HERV-FRD*), β-hCG (*CGbeta5*), and *GCM1* are shown. *GAPDH* was used as the housekeeping gene control. The results are presented as RQ with the minimum and maximum range (*n* = 5). (**D**) BeWo cells, treated with sicon, sip21, or mixed siRNAs against the coding region of p21 (sip21 #2) for 24 h, were incubated with forskolin or DMSO for another 48 h. Treated BeWo cells were stained for the fusion marker β-hCG (red) and DNA (DAPI, blue). Examples are shown. Scale: 10 µm. (**E**) BeWo cells were treated with sicon, sip21, or sip21 #2. After 24 h, the syncytin-2 promoter plasmid was transfected for 48 h. Luciferase assays of BeWo cells for syncytin-2 promoter activity is shown as mean value with SD (*n* = 3). Dot, square, and triangle show the individual data points of sicon, sip21 and sip21 #2, respectively. (**F**) Western blot analysis as transfection control. GAPDH was used as the loading control. Student’s *t*-test, * *p* < 0.05, ** *p* < 0.01, *** *p* < 0.001. ns, not significant.

Since the expression of p21 was decreased in fCTBs, a cell population ready to fuse into the STB of placental tissues from early-onset PE patients (Figure 3C, left graph), we focused on the role of p21 in cell differentiation and fusion. To look at possible mechanisms by which reduced p21 could cause defects in cell differentiation, total RNAs were extracted from BeWo cells depleted of p21 for RNA microarray analysis (Figure 6A, Table S1). Differently expressed genes were compared between BeWo cells treated with the control siRNA (sicon) and siRNA specifically targeting p21. The heatmap depicts genes with a *p*-value smaller than 0.05, and a fold change greater than 1 (red color code) and below 1 (blue color code), respectively (Figure 6A, left panel).

Interestingly, as reported for HTR cells depleted of p21 [25], the extracellular signal-regulated kinase 3 (ERK3), encoded by the gene *MAPK6*, is among the top three of altered genes and strongly reduced upon p21-depletion (hit number 1, *CDKN1A*). ERK3 is a distantly related member of the mitogen-activated protein kinase (MAPK) superfamily [48], which is known to be involved in cell differentiation [49]. Further analysis revealed that the fusion-related genes *HERV-FRD* (syncytin-2), *ERVWE1* (syncytin-1), *CGbeta5* (β-hCG), *KLF6* (Krüppel-like factor 6), and *GCM1* (glial cells missing transcription factor 1) were reduced (Figure 6A, right upper panel) upon p21 depletion (Figure 6A, right lower panel). This was corroborated for *HERV-FRD*, *CGbeta5*, and *GCM1* by quantitative PCR analysis with BeWo (Figure 6B) and JEG-3 cells (Figure 6C). To further address this issue, BeWo or JEG-3 cells depleted of p21 with two different siRNAs (sip21 and sip21 #2; Figure 6F and Figure S3G) were stimulated to fuse with forskolin up to 48 h. The amount of β-hCG, which is upregulated upon cell fusion and induced by forskolin [47], was reduced upon p21 depletion, visualized by immunofluorescence staining compared to the control cells (Figure 6D and Figure S3E). Moreover, the luciferase assay showed that reduced p21 significantly decreased the expression of syncytin-2 in a promoter-dependent manner in BeWo and JEG-3 cells, respectively (Figure 6E and Figure S3F).

## 4. Discussion

The cell cycle regulator p21 is a key player in various cellular events including cell differentiation, migration, stem cell maintenance, and gene transcription [24], processes important for placental development and altered in its pathogenesis. However, p21’s expression and roles in the placenta and its diseases are still contradictory, despite numerous studies and intensive work. To clarify these issues, in the present work, we collected early-onset PE, early-onset PE complicated by the HELLP syndrome, and late-onset PE placental samples, and their well-matched control tissues, and systematically examined the expression of p21 (*CDKN1A)* and its family members p27 *(CDKN1B)* and p57 *(CDKN1C)* as well as their roles in normal pregnancy and hypertensive disorders. We show here that these cell cycle regulators are highly expressed in first trimester placentas, while their expression generally decreases in the second and third trimester placenta at the protein as well as gene level. Notably, fCTBs, a special portion of CTBs ongoing to fuse into the STB, express high levels of p21 and its family members, which remain high throughout gestation. Importantly, the level of p21 is only reduced in fCTBs of early-onset PE placental tissues and its overall protein expression is decreased in early-onset PE complicated by the HELLP syndrome. These data strongly support the notion that early- and late-onset PE derive from distinct pathogenesis. Moreover, cell cycle regulators are decreased upon hypoxic conditions and depletion of p21 leads to reduced expression of fusion-related genes and an impaired fusion capacity of trophoblastic cells. In addition, the expression of these cell cycle regulators is dependent on the delivery mode, underscoring the importance of optimization and standardization of patient selection and placental collection for proper interpretation of existing data on cell cycle regulators.

We show that overall p21 was reduced in early-onset PE and early-onset PE complicated by the HELLP syndrome, whereas it was unaltered in late-onset PE, suggesting its potential involvement in the development of early-onset PE. Several studies provide insight into p21′s roles in PE and are, however, accompanied by inconsistency and divergence: one study reported that the gene and protein expression of p21 was increased in preeclamptic placentas [50], whereby another study showed that p21 expression was decreased via IHC staining [51]. In other investigations, its protein or gene expression was unchanged [52,53]. The contradictory expression of p21 in PE might be explained by the highly variable study design concerning gestational age without the differentiation in early- and late-onset PE or concomitant diseases like the HELLP syndrome, sample number, and mode of delivery. In further support of our results, a recent study showed a decrease in p21 in early-onset PE, whereas it was unchanged in late-onset PE at the gene and protein level [54]. The reduced p21 is proposed to lead to elevated Cdk2/cyclin E levels and reduced phospho-retinoblastoma protein (RB) critical for cell cycle progression, which hampered the differentiation and fusion process of CTBs to the STB [54]. 

PE, especially early-onset PE, is associated with constant hypoxia and oxidative stress of the placenta [17]. Interestingly, we show that hypoxia significantly decreased mRNA levels of *CDKN1A* (p21) and *CDKN1B* (p27) in diverse trophoblastic cell lines, organoids derived from first trimester placenta, and isolated primary cytotrophoblasts. Others reported that the p21 protein was downregulated in CTBs upon 2% oxygen supply for 72 h, which was 3.8-fold higher in normoxia cell extracts isolated from explants of anchoring villi of first trimester placenta [55]. Moreover, we observed that hypoxia also decreased the mRNA level of *TP53* (p53), the upstream regulator of p21. However, the p53 protein expression from whole cellular lysates was not affected. p53 downregulation was reported in the STB of primary isolated term trophoblasts upon 1% hypoxia for 24 h, associated with the induction of apoptosis [56]. Evaluation of nuclear p53 and its post-modifications is required to clarify whether p53 is responsible for reduced p21. Interestingly, transcriptional repressors of p21 like BCL6 (B cell lymphoma 6) [57] or TFAP2C [58] are known to be enhanced in PE [52,59]. In addition, the miRNA family miR-130 including miR-130a/b and miR-301a/b repressed *CDKN1A* in human pulmonary artery smooth muscle cells upon hypoxia [60]. Interestingly, miR-130a and miR-301a have been reported to be overexpressed in PE placenta [61]. These regulators may be responsible for the reduced expression of p21 under hypoxia, bridging its reduction to the pathogenesis of PE.

PE is associated with profound cellular dysfunctions including reduced differentiation and fusion ability [62], and p21 is of crucial importance for differentiation [24]. Indeed, p21 protein levels increased during spontaneous differentiation and fusion of term CTBs [63] as well as *CDKN1A* during differentiation of mouse trophoblast stem cells and fusion of BeWo cells upon forskolin treatment [64], whereas p53 levels were reduced during BeWo cell differentiation [65]. We report here that p21 levels were significantly decreased in fCTBs, a special portion of CTBs ongoing to fuse to the STB, in early-onset PE samples, suggestive of its involvement in CTB differentiation and fusion. Moreover, in early-onset PE complicated by the HELLP syndrome, the H-score of overall p21 was reduced, whereas phosphorylated-p21 was significantly elevated in fCTBs, possibly a compensatory mechanism in a crisis situation induced by the HELLP syndrome, by which the stress protein p21 is trying to be stabilized and possibly translocated to the cytoplasm, where it could exert its anti-apoptotic function but hardly affect the transcription of fusion-related genes. In fact, our data revealed that the fusion-related genes *HERV-FRD* (syncytin-2), *CGbeta5* (β-hCG), and *GCM1* were reduced upon p21 depletion. Importantly, decreased p21 significantly reduced the expression of syncytin-2 in a promoter-dependent manner in BeWo and JEG-3 cells. Indeed, p21 has been reported to interact as a co-activator with GCM1 to bind to the promoter region of syncytin-2, regulating its transcription [66]. Our findings are further supported by previous studies. Reduced p21 was reported to be involved in impaired fusion mediated by KLF6 silencing in BeWo cells [63]. Recently, it has been revealed that p21, but not its family member p27, coordinates trophoblast fusion contributing to G0 arrest and terminal differentiation [66]. Of significance, the reduced expression of the fusion-related genes/proteins syncytin-1 and syncytin-2 correlates with the severity of PE [67]. In sum, our data clearly suggest that reduced p21 in fCTBs compromises the expression of fusion-related genes, contributing to impaired differentiation and fusion of trophoblasts, an important hallmark of early-onset PE.

The studies concerning p27 and p57 in PE are rather limited: in one study, p27 and p57 were significantly increased in preeclamptic placentas [68]. However, in that study, the averaged gestational age of PE patients was lower than in the control group [68]. p57 has been reported to be important in trophoblast fusion [69,70], and in migration and invasion [71]. In early-onset PE samples, we observed a significant reduction in the percentage of p57 positive fCTBs, suggestive of its potential contribution to compromised CTB differentiation and fusion, possibly in collaboration with reduced p21, in early-onset PE placentas. Additionally, we detected a reduced p57 level in EVTs, indicative of a possible involvement in impaired cell motility, a hallmark of PE. In fact, the amount of p57 was also decreased in western blot analyses of early-onset PE placental samples, whereas we did not observe significant differences in p27 expression. Interestingly, mutant mice, defective only for the maternal p57 allele, displayed clinical manifestations of PE including proteinuria and elevated blood pressure [72]. However, a further study failed to reproduce these PE manifestations using the same mouse model, although mice exhibited placental abnormalities [73]. p57-mutant murine placentas demonstrated significant alterations of transcripts coding for a variety of molecules involved in blood pressure regulation, inflammation, and apoptosis [74]. Moreover, loss of p57 seems to be associated with placentomegaly due to unrestricted endoreduplication [75]. The role of p57 in placental development needs further investigation.

It has been shown that the co-expression of p53 and p21 can lead to cell cycle arrest via suppression of G2 phase and mitotic (M) genes by the DREAM (*D*imerization partner, *R*B-like proteins, *E*2Fs *A*nd *M*ulti-vulval B) multiprotein complex [76,77]. In cancer cells, loss of p21 reduced the DREAM binding to the cell cycle-dependent element (CDE) and cell cycle genes homology region (CHR) of the promoters, resulting in the expression of G2/M genes [77]. However, microarray analysis using BeWo cells depleted of p21 did not show an increase in G2/M genes, which could be attributed to their long cell doubling time [78]. Remarkably, it revealed other interesting candidate genes such as the elevated gene *TXNIP*, which encodes the protein thioredoxin interacting protein, a major regulator of the cellular redox signaling, protecting cells from oxidative stress [79]. Interestingly, TXNIP was expressed in the STB, CTBs, and endothelial cells of the human placenta, where its expression was increased in the second and third trimester [80]. TXNIP loss significantly increased p21 protein expression levels in ARPE-19 cells, inhibiting cell growth [81], whereas TXNIP stabilized p27 protein indirectly in fibroblasts [82]. Further investigations are required to study the relationship of p21 with these candidate genes and their functions in PE.

## 5. Conclusions

Taken together, we show that p21 and its family members p27 and p57 are highly expressed in the first trimester of pregnancy, pointing to their importance in early placental development. The expression of p21 in CTBs and the STB is further highlighted with 3D trophoblast organoids, which have been reported to mimic the placental villi structurally, phenotypically, metabolically, and endocrinologically [28,29]. Moreover, we report that p21 is reduced in early-onset PE fCTBs ongoing to fuse to the STB. This finding is further underscored by our observation that hypoxia reduces p21 in trophoblastic cells and organoids. Depletion of p21 decreases the expression of fusion-related genes such as syncytin-2 and impairs the fusion capability of trophoblasts, characteristic of PE. Moreover, the percentages of positive p57 fCTBs and EVTs are significantly reduced in early-onset PE placentas, indicating its potential involvement in CTB fusion and EVT migration. These data strongly suggest that p21 deficiency, in collaboration with reduced p57, is likely to contribute to the pathogenesis of early-onset PE.

## Figures and Tables

**Table 1:** Clinical information of patients for delivery mode analysis. Mean value ± standard deviation is shown. CS, caesarean section; eCS, emergency caesarean section after the onset of labor; VD, vaginal delivery; opVD, operative vaginal delivery; ns, not significant.

	*n*	Gestational Age (Weeks)	Body Mass Index (BMI)	Age	Birth Weight (g)
CS	5	38.2 ± 0.8	21.9 ± 1.4	28.4 ± 0.9	2960 ± 634
eCS	5	38.0 ± 1.0	21.5 ± 1.9	27.0 ± 2.2	3020 ± 370
VD	5	38.0 ± 1.0	21.7 ± 2.3	27.0 ± 1.9	3002 ± 302
opVD	5	38.0 ± 1.0	22.8 ± 1.0	26.4 ± 2.2	3008 ± 302
*p*-value		ns	ns	ns	ns

**Table 2:** Clinical information of patients with early-onset preeclampsia (ePE) and matched controls. Mean value ± standard deviation is shown. BP, blood pressure; n.d., not determined.

	*n*	Age	Gestational Age (Weeks)	Body Mass Index (BMI)	Birth Weight (g)	Systolic BP (mmHg)	Diastolic BP (mmHg)	Proteinuria (mg/24 h)
control	20	32.6 ± 4.6	29.7 ± 2.6	24.9 ± 3.9	1284 ± 710	118 ± 13	71 ± 11	n.d.
early-onset PE	20	32.4 ± 5.9	29.6 ± 2.6	25.7 ± 4.4	1072 ± 387	167 ± 22	102 ± 12	4153 ± 4569
*p*-value		0.876	0.330	0.256	0.114	0.00000011	0.0000038	

**Table 3:** Clinical information of patients with late-onset preeclampsia (PE) and matched controls. Mean value ± standard deviation is shown. BP, blood pressure; n.d., not determined.

	*n*	Age	Gestational Age (Weeks)	Body Mass Index (BMI)	Birth Weight (g)	Systolic BP (mmHg)	Diastolic BP (mmHg)	Proteinuria (mg/24 h)
control	10	30.9 ± 3.4	37.7 ± 1.3	23.4 ± 4.0	2914 ± 524	119 ± 7	73 ± 11	n.d.
late-onset PE	10	30.9 ± 2.8	37.7 ± 1.3	24.4 ± 2.0	2413 ± 461	153 ± 16	96 ± 13	1794 ± 1901
*p*-value		0.989	1.0	0.273	0.020	0.00034	0.00020	

**Table 4:** Clinical information of patients with early-onset preeclampsia (ePE) complicated by the HELLP (hemolysis, elevated liver enzymes, and low platelet count) syndrome and matched controls. Mean value ± standard deviation is shown. BP, blood pressure; n.d., not determined.

	*n*	Age	Gestational Age (Weeks)	Body Mass Index (BMI)	Birth Weight (g)	Systolic BP (mmHg)	Diastolic BP (mmHg)	Proteinuria (mg/24 h)
control	17	29.6 ± 9.1	30.2 ± 7.9	23.7 ± 6.7	1751 ± 920	109 ± 29	66 ± 20	n.d.
ePE + HELLP	16	31.4 ± 4.5	31.7 ± 2.4	25.3 ± 4.3	1389 ± 443	175 ± 21	107 ± 9	4815 ± 4856
*p*-value		0.589	0.164	0.973	0.0035	0.0000001	0.00000004	

**Table 5:** Clinical information of preeclamptic patients and matched donors, whose placentas were used for primary trophoblast isolation (term). Mean value ± standard deviation is shown. BP, blood pressure; n.d., not determined.

	*n*	Age	Gestational Age (Weeks)	Body Mass Index (BMI)	Birth Weight (g)	Systolic BP (mmHg)	Diastolic BP (mmHg)	Proteinuria (mg/24 h)
control	5	32.6 ± 5.3	40 ± 1.2	22.6 ± 3.7	3407 ± 448	114 ± 9	74 ± 11	n.d.
PE	5	34.1 ± 5	36.8 ± 4.0	23.7 ± 2.7	2462 ± 974	150 ± 11	97 ± 5	2763 ± 3459
*p*-value		0.666	0.123	0.604	0.084	0.00042	0.0031	

## Data Availability

The datasets generated and/or analyzed during the current study are available from the corresponding author on reasonable request.

## Supplementary Materials

The following are available online at https://www.mdpi.com/article/10.3390/cells10092214/s1, Figure S1: Characterization of long-term trophoblast organoid culture, Figure S2: Expression of cell cycle regulators in late-onset PE, Figure S3: Characterization of primary cytotrophoblasts. Figure S4: Raw data of all western blots. Table S1: Data from whole gene-expression array with a *p*-value smaller than 0.05.
