# Supplementary material for: Functional Analysis of p21Cip1/CDKN1A and Its Family Members in Trophoblastic Cells of the Placenta and Its Roles in Preeclampsia
Source: Cells. 2021 Aug 27;10(9):2214. doi: 10.3390/cells10092214 (PMC8465116; doi:10.3390/cells10092214)
Supplement: Supplementary file 1 [file cells-10-02214-s001.zip › Supplementary Materials.pdf]

Supplementary Materials

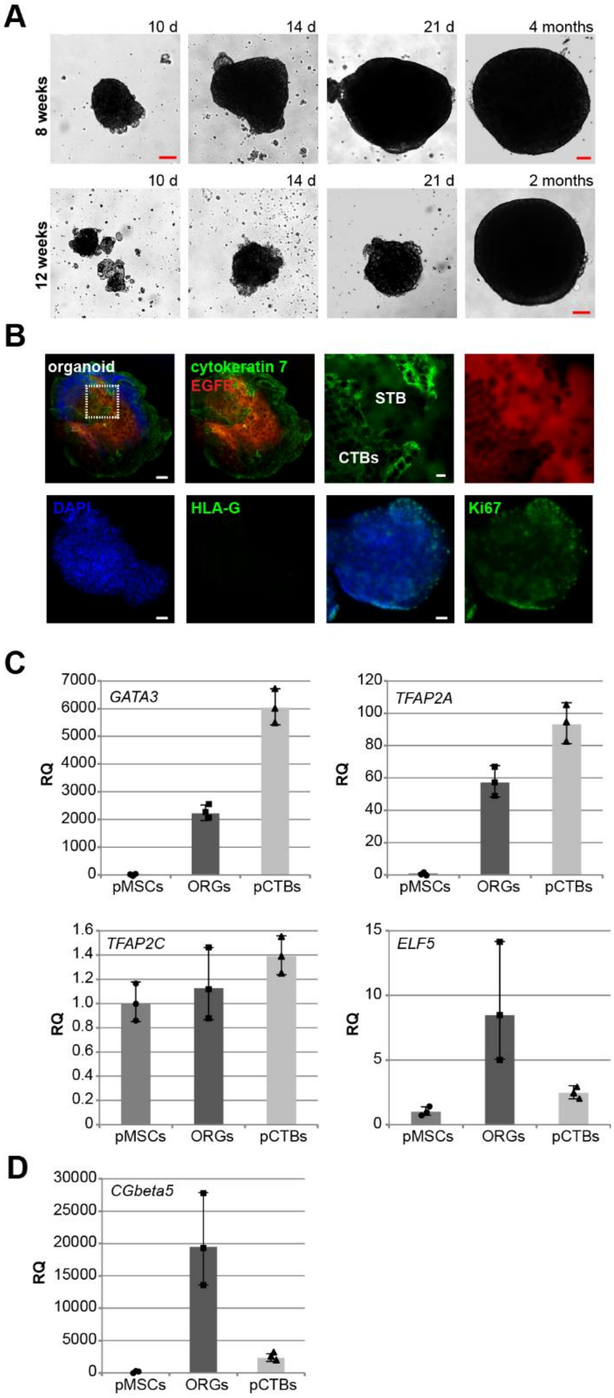

Figure S1. **Characterization of long-term trophoblast organoid culture.** (**A**) Bright-field images of trophoblast organoids derived from trophoblasts of 8 weeks (upper panels) and 12 weeks of gestation (lower panels) at indicated time points. Scale: 100 µm. (**B**) Upper panel: IHC-IF images of trophoblast organoids stained with cytokeratin 7 (green, CTB marker), EGFR (red, STB marker) and DAPI (DNA, blue). Scale, 1st picture: 50 µm; inset scale (2nd to 4th picture): 10 µm. Lower panel: IHC-IF images of trophoblast organoids stained with HLA-G and DAPI (1st and 2nd image), or Ki67 and DAPI (3rd and 4th image), scale: 50 µm. (**C** and **D**) Gene analysis of placental mesenchymal stem/stromal cells (pMSCs), organoids (ORGs) and isolated primary first trimester cytotrophoblasts (pCTBs). The results are presented as RQ with minimum and maximum range. *TBP* was used as housekeeping gene control. (**C**) The mRNA levels of *GATA3*, *TFAP2A*, *TFAP2C* and *ELF5* are shown. (**D**) The mRNA levels of β-hCG (*CGbeta5*) are displayed. *TBP* was used as endogenous control.

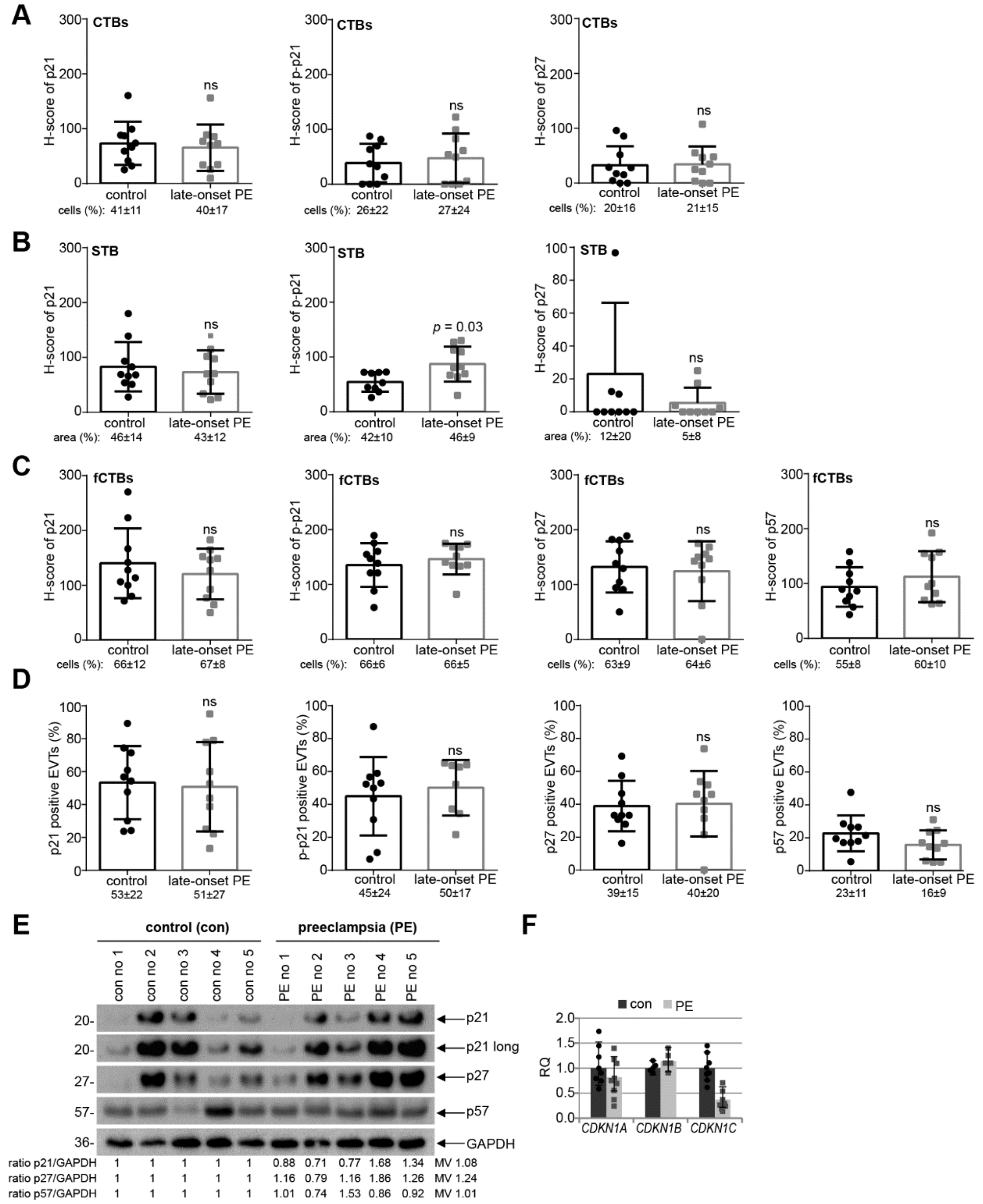

Figure S2. Expression of cell cycle regulators in late-onset PE. (**A**-**C**) Quantification of cell cycle regulators in placental tissue sections from healthy donors (control, n = 10) and patients with late-onset PE (n = 10) using the H-score method. The results are presented as bar and scatter plots showing the mean value with SD. The percentage of positive stained cells/area is shown under the graphs. (**A**) H-score of p21 (left panel), p-p21 (middle panel) and p27 (right panel) for CTBs. (**B**) H-score of p21 (left panel), p-p21 (middle panel) and p27 (right panel) for the STB area. (**C**) H-score of p21 (left panel), p-p21 (second panel), p27 (third panel) and p57 (right panel) for fCTBs. (**D**) Quantification of p21 positive (left panel), p-p21 positive (second panel), p27 positive (third panel) and p57 positive EVTs (right panel) in %. (**E**) Western blot analysis with extracts from placental tissues is shown. GAPDH served as loading control. (**F**) The relative amount of the gene levels was analyzed from placental tissues: *CDKN1A* (p21), *CDKN1B* (p27) and *CDKN1C* (p57). The results are presented as relative quantification (RQ) with minimum and maximum range. *TBP* was used as endogenous control. Paired Student's *t*-test or Wilcoxon-test was used for statistical analysis. CTBs, cytotrophoblasts; fCTBs, cytotrophoblasts ongoing to fuse; STB, syncytiotrophoblast; EVT, extravillous cytotrophoblasts; no, number; MV, mean value.

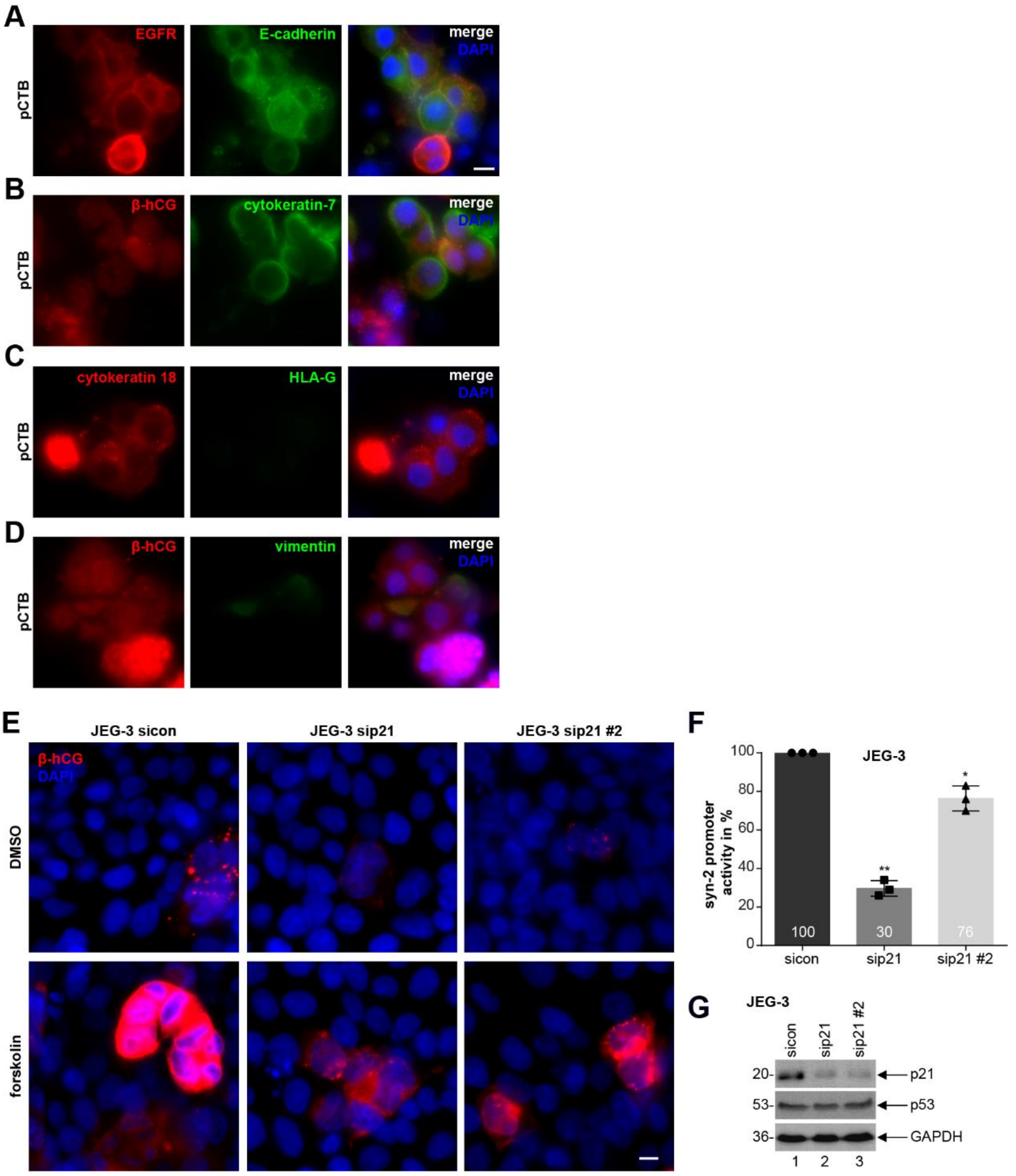

Figure S3. Characterization of primary cytotrophoblasts. Isolated primary cytotrophoblasts (pCTBs) were characterized by immunofluorescence staining for (**A**) EGFR (red) and E-cadherin (green), (**B**) for β-hCG (red) and cytokeratin 7 (green), (**C**) for cytokeratin 18 (red) and HLA-G (green, negative marker), and (**D**) for β-hCG (red) and vimentin (green, negative marker). Scale: 10 µm. (**E**) JEG-3 cells, treated with sicon, sip21 or mixed siRNAs against the coding region of p21 (sip21 #2) for 24 h, were incubated with forskolin or DMSO for another 48 h. Treated JEG-3 cells were stained for the fusion marker β-hCG (red) and DNA (DAPI, blue). Examples are shown. Scale: 10 µm. (**F**) JEG-3 cells were treated with sicon, sip21 or sip21 #2. After 24 h, the syncytin-2 promoter plasmid was transfected for 48 h. The results of syncytin-2 promoter activities are shown from luciferase assays of treated JEG-3 cells as mean value with SD (n = 3). Dot, square, and triangle show the individual data points of sicon, sip21 and sip21 #2, respectively. (**G**) Western blot analysis as transfection control. GAPDH was used as loading control. Student's *t*-test, * $p < 0.05$, ** $p < 0.01$.

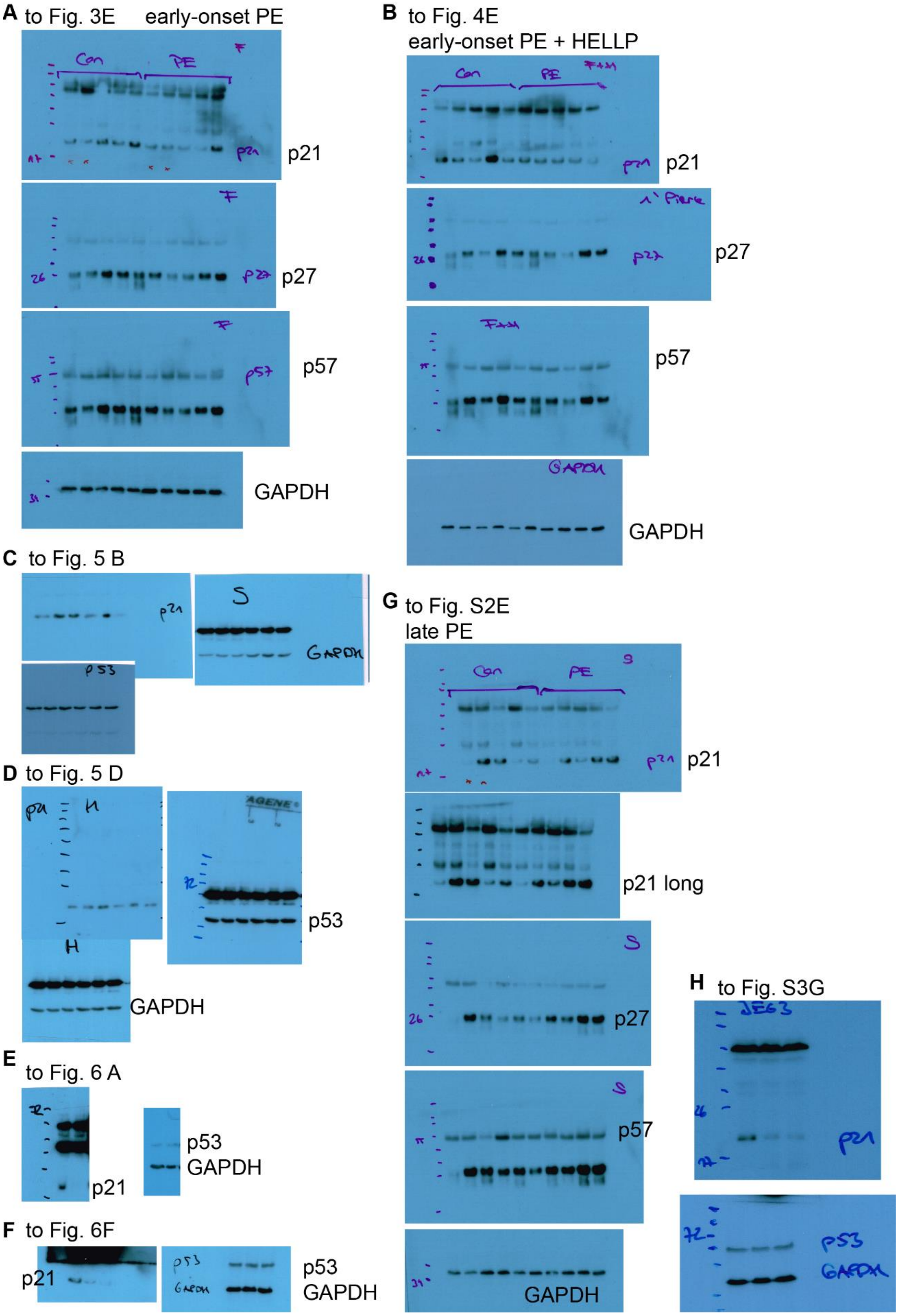

Figure S4. Raw data of all western blots. (**A**) Raw data to Figure 3E. (**B**) Raw data to Figure 4E. (**C**) Raw data to Figure 5B. (**D**) Raw data to Figure 5D. (**E**) Raw data to Figure 6A. (**F**) Raw data to Figure 6F. (**G**) Raw data to Figure S2E. (**H**) Raw data to Figure S3G.
